# Normal tissue radioprotection by amifostine via Warburg-type effects

**DOI:** 10.1038/srep30986

**Published:** 2016-08-10

**Authors:** Michael I. Koukourakis, Alexandra Giatromanolaki, Christos E. Zois, Dimitra Kalamida, Stamatia Pouliliou, Ilias V. Karagounis, Tzu-Lan Yeh, Martine I. Abboud, Timothy D. W. Claridge, Christopher J. Schofield, Efthimios Sivridis, Costantinos Simopoulos, Savvas P. Tokmakidis, Adrian L. Harris

**Affiliations:** 1 Department of Radiotherapy/Oncology, Democritus University of Thrace, Alexandroupolis 68100, Greece; 2 Department of Pathology, Democritus University of Thrace, Alexandroupolis 68100, Greece; 3 Cancer Research UK, Molecular Oncology Laboratories, Weatherall Institute of Molecular Medicine, University of Oxford, Oxford, UK; 4 The Chemistry Research laboratory, Mansfield Road, Oxford, OX1 3TA, UK; 5 Laboratory of Experimental Surgery, University Hospital of Alexandroupolis, Democritus University of Thrace, Alexandroupolis, Greece; 6 Department of Physical Education and Sports Science. Democritus University of Thrace, Komotini, Greece

## Abstract

The mechanism of Amifostine (WR-2721) mediated radioprotection is poorly understood. The effects of amifostine on human basal metabolism, mouse liver metabolism and on normal and tumor hepatic cells were studied. Indirect calorimetric canopy tests showed significant reductions in oxygen consumption and of carbon dioxide emission in cancer patients receiving amifostine. Glucose levels significantly decreased and lactate levels increased in patient venous blood. Although amifostine *in vitro* did not inhibit the activity of the prolyl-hydroxylase PHD2, experiments with mouse liver showed that on a short timescale WR-1065 induced expression of the Hypoxia Inducible Factor HIF1α, lactate dehydrogenase LDH5, glucose transporter GLUT2, phosphorylated pyruvate dehydrogenase pPDH and PDH-kinase. This effect was confirmed on normal mouse NCTC hepatocytes, but not on hepatoma cells. A sharp reduction of acetyl-CoA and ATP levels in NCTC cells indicated reduced mitochondrial usage of pyruvate. Transient changes of mitochondrial membrane potential and reactive oxygen species ROS production were evident. Amifostine selectively protects NCTC cells against radiation, whilst HepG2 neoplastic cells are sensitized. The radiation protection was correlates with HIF levels. These findings shed new light on the mechanism of amifostine cytoprotection and encourage clinical research with this agent for the treatment of primary and metastatic liver cancer.

Amifostine (WR-2721) is a cytoprotective agent used in radiotherapy for protection against xerostomia^1^. There is also strong evidence that amifostine reduces radiation induced mucositis and pneumonitis^2^. Following rapid hydrolysis by the alkaline phosphatases of normal vessels, the active metabolite of amifostine, WR-1065, enters into the normal cells where it is proposed to act as a potent free radical scavenger, so reducing the DNA damage^3^. Its intracellular conversion to the disulphide WR-33278, which has structural similarities with the polyamines, promotes DNA damage repair^4^. Both thiol WR-1065 and the disulfide derived from it, WR-33278, have the ability to remove platinum adducts from DNA^5^, in accord with clinical data showing protection against cis-platin related normal tissue toxicities^2^.

Amifostine treatment has multiple effects, which all converge on the protection of cells against cytotoxic DNA damage induced by ionizing radiation or DNA-damaging chemotherapeutic agents. This protection is selective for normal tissues, since several differences in the biology of tumoral tissues prevent either the accumulation of amifostine in cancer cells or its cytoprotective function^6^. The complex cytoprotective pathways exploited by amifostine may also involve interference with genes involved in cell cycle regulation and apoptosis^7^. Overall, the interactions of amifostine with normal and cancer cellular and tissue biology are poorly understood.

The effect of amifostine on cellular metabolism was recognized in early experiments. Using microcalorimetric studies, Purdie *et al*., showed that incubation of cells with WR-1065 results in heat production that lasts for at least 90 minutes^8^. This heat, possibly related to the oxidation of WR-1065 to disulfides, leads to a rapid consumption of the intracellular oxygen. In principle, the resultant intracellular hypoxia could be corrected *in vivo* by increasing oxygen extraction from the blood; however, this does not occur. Many years ago, Glover *et al*. observed that, in contrast to expectation, the mean venous oxygen tension and the hemoglobin oxygen saturation increases following amifostine administration^9^. Although this observation suggests that decreased oxygen release to normal tissues may contribute to amifostine cytoprotective pathways, the finding was not followed up. In a study of ours, the reduced consumption of blood oxygen, shown by the sharp rise of venous blood oxygen tension and increased haemoglobin saturation, was confirmed 20 minutes after intravenous administration of amifostine^10^. This effect was transient, because 60–120 min after amifostine administration oxygen levels was restored to the pre-treatment levels.

In the current study we investigated the effects of amifostine on the basal metabolic rate (BMR) of patients with breast cancer, providing evidence of a shift of the normal tissue metabolism to glycolysis and reduced oxygen consumption, typical of the Warburg effect, which may be a component of the complex cytoprotective pathways exploited by amifostine. This metabolic shift was confirmed in mouse and in *in vitro* experiments, by induction of enzymes involved in glycolysis and blockage of mitochondrial pyruvate usage. This new mechanism represents a first-in-class effect with implications for reducing O_2_ consumption and inducing cytoprotective pathways depend on HIF, in other diseases and preventing redox injury.

## Results-Discussion

### Effect of amifostine on human metabolism

**Figure 1:**
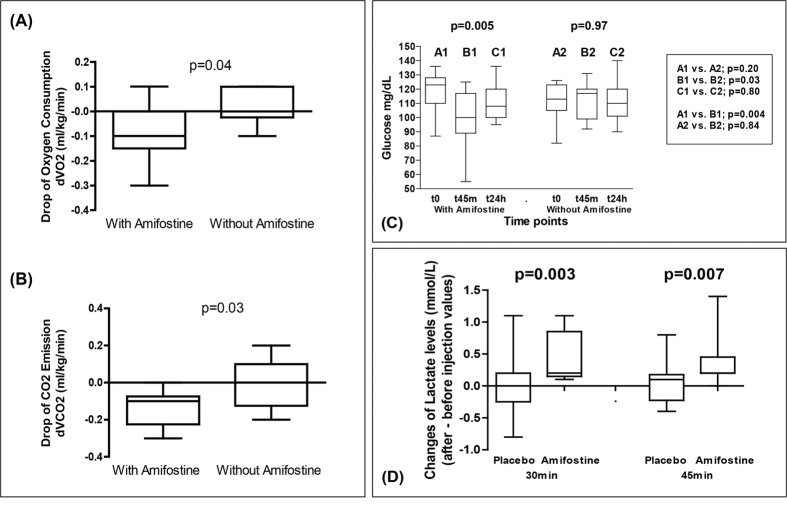
(**A,B**) Decrease in oxygen consumption and in carbon dioxide emission from breast cancer patients (indirect calorimetric canopy metabolism assessment) injected with amifostine vs. placebo (d = values after – values before injection). (**C**) Time course changes of the glucose levels in the venous blood of breast cancer patients injected with amifostine vs. placebo. (**D**) Time course changes of the lactate levels (final – initial value) in the venous blood of breast cancer patients injected with amifostine vs. placebo. Boxes and whiskers show min and max value, mean value and 25%/75% percentile values. One way ANOVA non parametric analysis (Kruskal-Wallis test) and the Wilcoxon matched pairs test were used to compare groups with continuous variable data.

We first investigated whether administration of amifostine to patients who were due to have radiotherapy for breast cancer to the primary site, produced changes in basal metabolism rates. Using an indirect calorimetric canopy metabolism assay^11^ we observed a sharp reduction of the oxygen consumption in patients injected subcutaneously with 500 mg of amifostine (the median dVO_2_ assessed in ml/kg per min was −0.10 ± 0.04 in pts receiving amifostine vs. 0.10 ± 0.02 in controls; p = 0.04; Fig. 1A). The production of carbon dioxide (CO_2_) also decreased (median dVCO_2_ was −0.13 ± 0.03 in pts receiving amifostine vs. −0.01 ± 0.04 in controls; p = 0.03; Fig. 1B).

These results are indicative of a suppression of the Krebs cycle (reduced oxygen consumption and CO_2_ production due to reduced oxidative usage of glucose/pyruvate). In parallel with these findings, 45 min following amifostine administration, the initial glucose levels (118 ± 15 vs. 113 ± 12 mg/dL; p = 0.20 for amifostine and placebo groups, respectively), dropped significantly in the amifostine group (to 98 ± 19 mg/dL; p = 0.004), while the levels in the placebo group were maintained at a stable level (111 ± 13 mg/dL; p = 0.84). The glucose levels returned to pretreatment values at 24 hours in both groups (Fig. 1C). The one-way ANOVA test showed a significant change of glucose levels in the amifostine group (p = 0.005) but no change in the placebo group (p = 0.97).

Taking into account the above clinical results, we hypothesised that amifostine stimulates glucose consumption followed by pyruvate transformation to lactate, to compensate for energy loss that occurring following from Krebs cycle suppression. The lactate pathway is less efficient than the Krebs cycle, in terms of ATP production form pyruvate, and does not release CO_2_, compatible with the decreased emission observed in our patients^12^. Despite the uncertainty in the rate of lactate recycling expected among patients, a significant change in lactate levels was noted following amifostine injection (Fig. 1D). The mean increase of lactate levels at 30 min was 0.54 ± 2.7 mg/dL (0.06 ± 0.3 mmol/L) following placebo vs. 3.69 ± 2.7 mg/dL (0.41 ± 0.3 mmol/L) following amifostine injection (p = 0.003). The mean increase of lactate levels at 45 min was 0.72 ± 2.7 mg/dL following placebo vs. 3.24 ± 2.7 mg/dL following amifostine injection (0.08 ± 0.3 vs. 0.36 ± 0.3 mmol/L; p = 0.007). The cause of such a shift in lactate and glucose concentrations is unlikely to be due to extra-cellular mechanisms, because amifostine does not interfere with oxygen dissociation from hemoglobin, nor does it change the pH in red cells^9^. Regulation by amifostine of proteins involved in glycolysis was therefore postulated.

### Mouse liver and *in vivo* metabolic effect of amifostine

**Figure 2:**
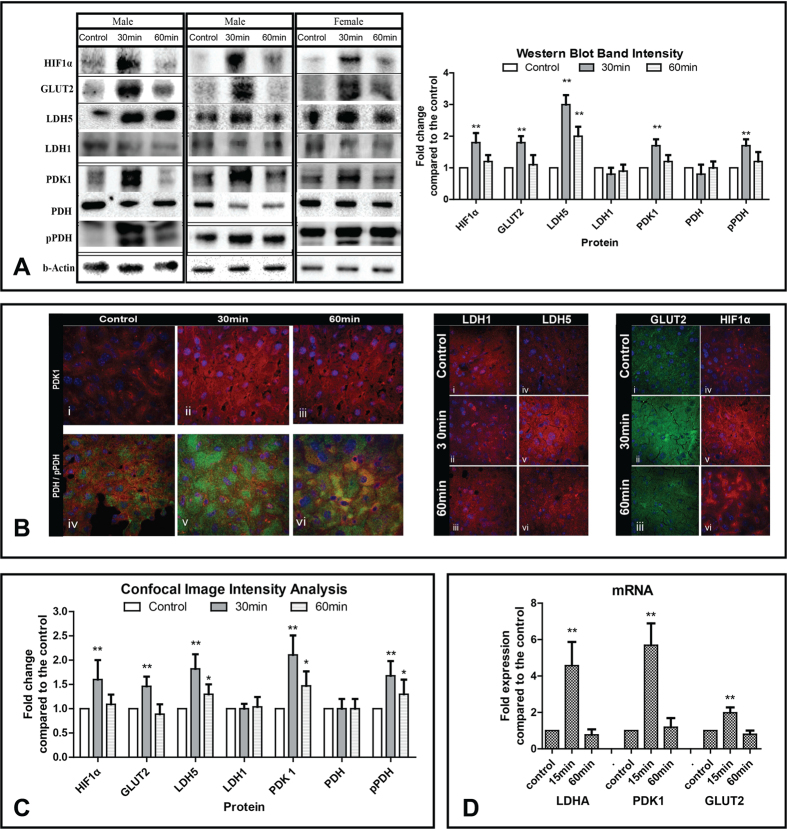
(**A**) Western blot images and band densitometry analysis of levels of proteins involved in anaerobic metabolism, as assessed in mouse liver before and after administration of amifostine. Bars show standard deviation and asterisks refer to p-values (*p < 0.05, **p < 0.001). (**B**) Confocal immunofluorescent microscopy after staining with anti-PDK1/red antibody showing increased cytoplasmic expression in mouse hepatocytes from 0 (i) to 30 min (ii) and regression thereafter (iii). Double immunostaining with PDH/red and phosphorylated pPDH/green (iv), showed an intensification of the expression of the inactive pPDH form of the enzyme 30 min (v) following amifostine injection and trend for restoration of normal PDH levels at 60 min (vi). Confocal immunofluorescent microscopy after staining for LDH1/red showed stable levels of expression in mouse hepatocytes (i,ii,iii). In contrast, LDH5/red expression was sharply induced 30 min following amifostine injection and decreased thereafter (iv, v, vi). Similar patterns were noted for GLUT2 expression (i,ii,iii) and for HIF1α expression (iv,v,iv). (**C**) Analysis of the fluorescence intensity of confocal microscopy images (from five representative tissue areas for each staining). Bars show standard deviation and asterisks refer to p-values (*p < 0.05, **p < 0.001). (**D**) mRNA expression levels of LDHA, PDK1 and GLUT2 (three mice for each time point) following exposure to amifostine, as measured with quantitative RT-PCR. Bars show standard deviation and asterisks refer to p-values (**p < 0.001).

To investigate this hypothesis, we studied the *in vivo* metabolic effect of amifostine on normal mouse liver. Mice were injected with amifostine and their livers were collected before, 30 and 60 min following subcutaneous injection of amifostine (200 mg/kg corresponding to 35% about of the maximum tolerable dose in mice). Several glycolytic enzymes were sharply induced at 30 min. Figure 2A shows western blot analysis and band intensity analysis of the enzymes involved in pyruvate metabolism in mouse liver. There was an increase of hypoxia inducible factor HIF1α, glucose transporter GLUT2, lactate dehydrogenase 4M (LDH5), pyruvate dehydrogenase kinase PDK1 and the phosphorylated form of pyruvate dehydrogenase pPDH (E1-alpha subunit phosphorylated at serine 293), while lactate dehydrogenase 4H (LDH1) remained stable. LDH1 is well recognised not to respond to HIF1 in contrast to LDH5. PDH is regulated by phosphorylation by a HIF dependent kinase, PDK1 (which also went up on the blots at 30 mins), so only the phosphorylation of the protein changed, not the amount. The levels of the enzymes decreased, but were still above basal and control levels, at 60 min post-injection.

Confocal immunofluorescent microscopy of liver tissue confirmed the western blot findings. PDK1 expression was intensified following amifostine injection and, in double immunostaining, phosphorylated (inactive) pPDH showed a marked accumulation at 30 min, which regressed at 1 h (Fig. 2B). An increase of ΗΙF1α, GLUT2, LDH5 was noted at 30 min, while LDH1 expression remained stable or declined following amifostine exposure (Fig. 2B). Fluorescence intensity analysis of images is shown in Fig. 2C.

GLUT2 is the main glucose transporter in adult hepatocytes^13^, and its increase would facilitate an acceleration of glucose uptake, which in humans would explain the drop of glucose levels found in breast cancer patients 45 min after injection of amifostine. The fate of the increased intracellular glucose is likely to be increased glycolysis, based on the increase of PDK1 and its substrate phosphorylated-PDH. These findings suggest a profound suppression of mitochondrial respiration by amifostine, as PDH is no longer available to catalyse the transformation of pyruvate to Acetyl-CoA, the key substrate of the Krebs cycle.

Changes induced by amifostine in the mRNA expression profiles of PDK1, LDHA and GLUT1 genes in mouse liver were further studied using real-time quantitative PCR. Figure 2D shows that PDK1 gene expression are increased 5.6-fold 15 minutes after amifostine injection (p < 0.001). A significant increase (p < 0.001), 4.5-fold, was observed in expression of LDHA 15 minutes after injection of amifostine, while the level of GLUT2 gene expression are 2-fold higher than control at the same time point (p < 0.001). The expression of genes returned to control levels 60 minutes after the amifostine injection. The mRNA levels of these genes paralleled the protein levels, after exposure to amifostine.

The protein changes that occured in target tissues, matched the short half-lif of amifostine. They are more rapid than reported in tissue culture. These studies were done in intact whole organs *in vivo*, with high metabolic activity but low proliferation and we presume they result in the different kinetics *in vivo* which have not been studied before. We hypothesise that high mTOR activity resulted in more efficient translation of induced mRNA, but higher proteasomal activity resulted in more rapid clearance, compared to previous cell line work.

### *In vitro* metabolic effect on hepatocyte and hepatoma cells

**Figure 3:**
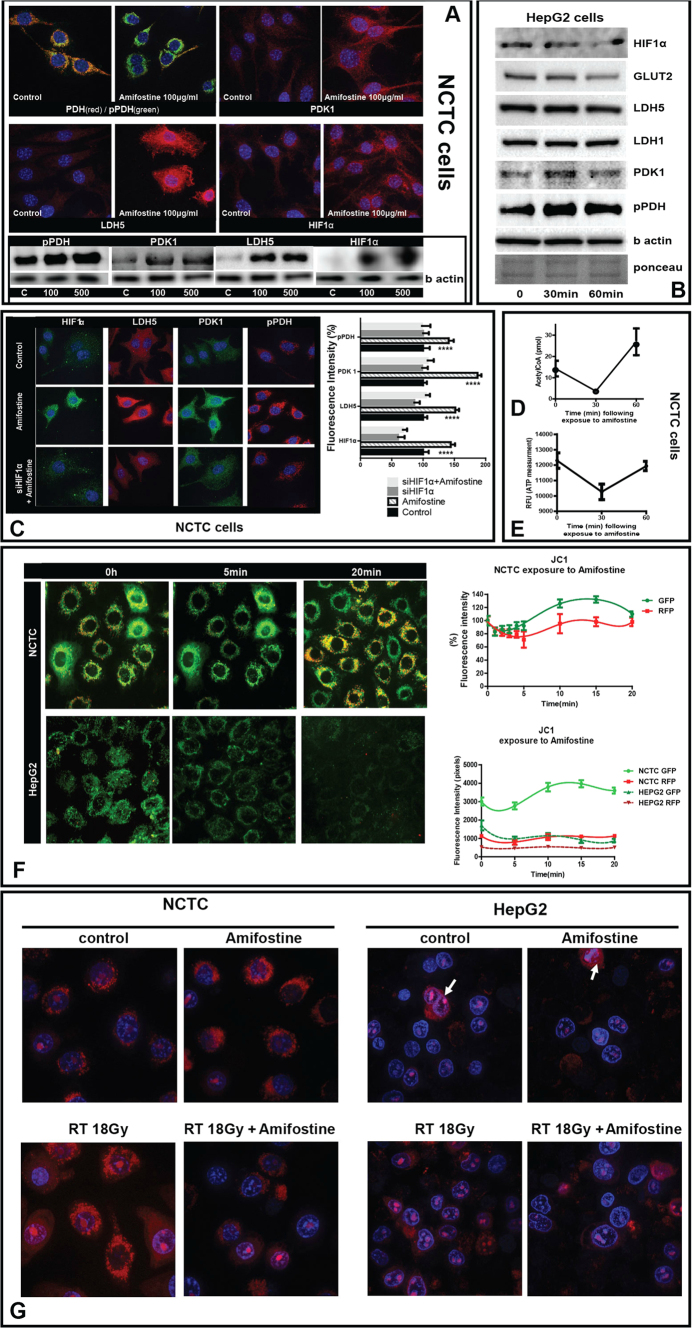
(**A**) Confocal microscopy of NCTC liver cells before and after exposure to amifostine (100 μg/ml), for double PDH(red)/phosphoPDH(green), phosphoPDH(red), LDH5 (red) and HIF1α (red) expression. Western blot bands following exposure to 100 and 500 μg/ml of amifostine at 30 min is also shown. (**B**) Western blot expression of phosphoPDH, PDK1, LDH5 and HIF1α in hepatoma HepG2 cells, at 0, 30 and 60 min following exposure to 100 μg/ml of amifostine. (**C**) Confocal microscopy of NCTC liver cells before and after exposure to amifostine (100 μg/ml), for HIF1α (green), LDH5 (red), PDK1 (green) and phosphoPDH(red) expression with and without silencing of the HIF1α gene. (**D**) Acetyl-CoA levels (pmol) in NCTC cells at 0, 30 and 60 min following exposure to 100 μg/ml of amifostine. (**E**) ATP levels (pmol) in NCTC cells at 0, 30 and 60 min following exposure to 100 μg/ml of amifostine. (**F**) Time course recording of NCTC and HepG2 cell mitochondrial membrane potentials as assessed with the JC1 method and confocal imaging (0–20 minutes), showed a rapid transient reduction of green (monomer) and red (aggregate) forms of the dye that was subsequently restored to normal levels. (**G**) Mitochondrial ROS (mtROS) production by NCTC and HepG2 cells, after exposure to 18Gy of ionizing radiation with and without pre-incubation with amifostine, showing a strong effect of amifostine in normal NCTC cells. mtROS were low in hepatoma HepG2 cells compared to NCTC hepatocytes and were increased only in dividing neoplastic cells.

*In vitro* studies were undertaken to investigate the mechanisms of the effect of amifostine, using normal NCTC hepatocytes (National Collection of Types Cultures). Incubation of normal hepatocytes (NCTC cells) with amifostine (100–500 μg/ml) for 1 h, resulted in a sharp increase of pPDH, PDK1, LDH5 and HIF1α, as shown by confocal microscopy and western blot analyses (Fig. 3A). In contrast, amifostine had no effect on the expression of these proteins in a human hepatoma cell line (HepG2) (Fig. 3B). Silencing HIF1α in NCTC cells blocked the metabolic response of cells to amifostine, which were unable to increase pPDH and LDH5 expression (Fig. 3C).

In order to assess the effect of amifostine on the Krebs cycle, changes in the intracellular acetyl-CoA levels were recorded after 1 h exposure of NCTC cells to 100 μg/ml of amifostine. At 30 min there was a sharp drop of the acetyl-CoA levels, which were restored at 60 min to levels higher than the initial ones (Fig. 3D). This latter effect further supports the proposal of a suppressed aerobic mitochondrial function, due to low Acetyl-CoA levels, as a result of amifostine induced PDH phosphorylation and blockage of pyruvate transformation to acetyl-CoA. Measurement of ATP at the same time points showed a sharp reduction in intracellular ATP at 30 min, compatible with the switch to anaerobic ATP production, a pathway known to be far less ATP productive than oxidative phosphorylation. The levels returned to normal at 60 min (Fig. 3E).

We assessed the effect of amifostine on mitochondrial membrane potentials, using the JC1 (tetraethylbenzimida-zolylcarbocyanine iodide) method. The accumulation kinetics of green (monomer) and red (aggregates) fluorescence in mitochondria was assessed using confocal microscopy of NCTC cells, after exposure to 500 μΜ of amifostine. Serial images confirmed a rapid drop of both green and red fluorescence, one minute after exposure, an effect that was restored to normal at 20 minutes, after a small period of a rebound increase. This showed that amifostine affected mitochondrial membrane potential, associated with a short lasting reduced oxygen flux and consumption (Fig. 3F).

Similar experiments were performed using the HepG2 hepatoma cell line. The steady state JC1 green staining was far lower than in NCTC cells, and the red staining was practically absent (Fig. 3F), showing that the neoplastic cells have disturbed mitochondrial membrane potentials supporting a reduced oxygen usage by the cancer mitochondria. Amifostine further reduced the JC1 green staining, an effect that was sustained throughout the 20 minutes of recording. This shows that amifostine enters hepatoma cells and further affects the already altered mitochondrial potential in hepatoma cells, which unlike normal cells, shows a prolonged effect. We hypothesised that the already malfunctioning mitochondria result in glycolytic metabolism in hepatoma cells, so that a further decrease of mitochondrial potential induced by amifostine and an eventual further suppression of oxygen entrance or utilization may have no additional metabolic effect, consistent with Western blot and image analysis.

The mitochondria mtROS production was also studied using confocal microscopy (mito-SOX Red mitochondrial superoxide indicator for live cell imaging). Irradiation of NCTC cells resulted in increased production of mtROS as evident by the increased expression of the dye in the mitochondria. Pretreatment with amifostine resulted in lack of a detectable increase in mtROS levels (Fig. 3G). HepG2 hepatoma cells on the other hand had very low levels of mtROS expression, while dividing cells were the only ones showing a strong mtROS production (Fig. 3G). Irradiation resulted in a slight increase of mtROS levels and amifostine pretreatment reduced this already minor effect. This shows that amifostine suppresses ROS production from mitochondria in irradiated normal hepatocytes, protecting eventually against mtROS mediated DNA damage. Such an effect is of far less importance in hepatoma cells.

This blockage of mitochondrial respiration in normal hepatocytes, resulted in up-regulation of the LDHA gene, which increases the levels of LDH isoenzymes involved in the transformation of pyruvate to lactate, a process that releases ATP to compensate for the Krebs cycle suppression. Indeed, the LDH5 isoenzyme, composed of 4 LDHA encoded M-subunits^14^, was increased in the liver of mice treated with amifostine and in NCTC cells, but not in the hepatoma cells.

This type of response can explain the increased lactate levels found in the venous blood of breast cancer patients. In contrast, the LDH1 isoenzyme levels, composed of 4 H-subunits encoded by the LDHB gene, remained unaltered. This later isoenzyme favors the reverse transformation of lactate to pyruvate, in tissues demanding pyruvate for oxidative phosphorylation, such as the heart. It seems, therefore, that amifostine triggers anaerobic metabolism through PDK gene overexpression and subsequent repression of the mitochondrial respiration. HIF1α was up-regulated by amifostine. Both PDK and LDHA are regulated by HIF1α^15^,^16^, and silencing of HIF1α blocked the activity of amifostine on PDK1 and PDH phosphorylation.

These results, arising from both human and mouse experiments, strongly support the proposal that changes in mitochondrial function producing a transient glycolytic switch, are an important component of the cytoprotection mechanism manifested by amifostine. Cancer cells are well known to have a predilection to aerobic glycolysis, the Warburg effect^17^. The hypoxia inducible factors HIFs and the down-stream target gene LDHA (which catalyses the transformation of pyruvate to lactate), are highly up-regulated in many tumors^18^. This hypoxic response may be an important reason for cancer cell radio-resistance^19^.

In contrast, normal cells rely on oxygen for ATP production and normally have a low content of HIFs and LDHA^17^,^18^. A rapid shift of normal tissue metabolism to glycolytic pathways induced by amifostine may simulate the endogenous hypoxia of tumor cells, reducing normal tissue susceptibility to radiation. In a previous study we noted an increase of HIF1α expression in normal rat tissue following amifostine administration, an effect also confirmed in cell cultures^10^. Allalunis-Turner *et al*. noted that administration of amifostine at maximally radioprotective doses significantly increased the binding of the hypoxia marker [3H]misonidazole to bone marrow cells, suggesting that hypoxia may contribute to amifostine mediated cytoprotection^20^.

### Amifostine and Prolyl hydroxylase 2

**Figure 4:**
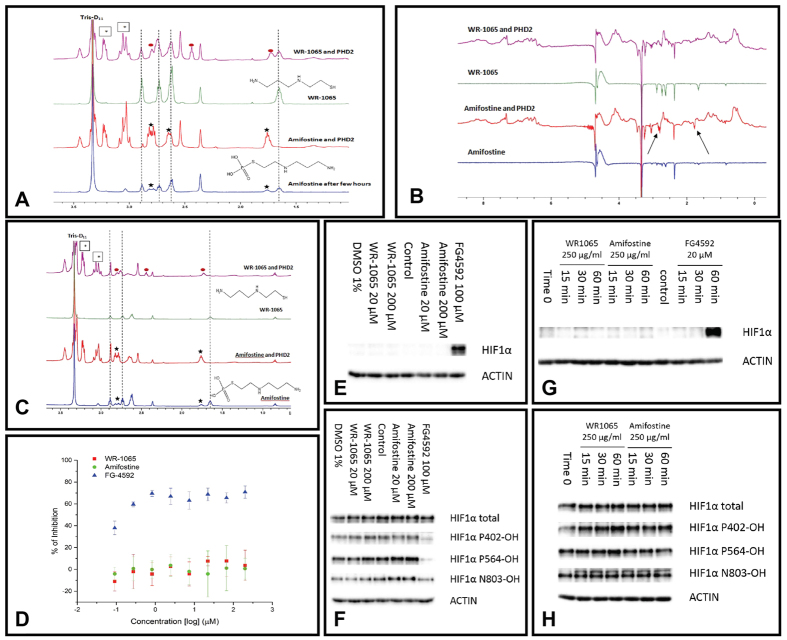
(**A**) **Single-concentration qualitative binding screening by** ^**1**^**H NMR.** Assay: 500 μM of compound (amifostine/WR-1065), 200 μM Zn(II), 50 μM *apo*-PHD2, where necessary, buffered with 50 mM Tris-D_11_ (pH 7.5), 0.02% NaN_3_ in 90% H_2_O/10% D_2_O. Peaks for amifostine: black stars, dephosphorylated amifostine/WR-1065: dashed lines, disulfide WR-1065 (WR-33278): red circles, protein sample peaks (e.g. glycerol): boxed stars. Amifostine reacts giving thiol WR-1065. No evidence for phosphorylated amifostine binding to PHD2 is apparent. Line broadening of WR-1065 peaks, characteristic of weak binding. (**B**) **Screening by wLOGSY NMR.** Assay: 500 μM amifostine or WR-1065, 200 μM Zn(II), 50 μM *apo*-PHD2, if needed buffered as above in 90% H_2_O/10% D_2_O. Results imply that amifostine doesn’t bind to PHD2 (compound peaks are highlighted by arrows) within detection limits; WR-1065 shows evidence of weak binding. (**C**) ^**1**^**H CPMG NMR screening of equimolar mixtures.** Assay: 100 μM amifostine/WR-1065, 200 μM Zn(II), 100 μM *apo*-PHD2; if needed buffered as above in 90% H_2_O /10% D_2_O. For WR-1065 partial reduction in peak intensity is observed, characteristic of weak binding, with the appearance of a new species, WR-33278. (**D**) Inhibition of the PHD2 catalytic domain of PHD2 (residues 181–426), assayed by AlphaScreen method, shows no evidence of inhibition by amifostine/WR-1065 (to 200 μM). FG-4592 is a positive control. Conditions: 5 nM PHD2_181–426_, 20 mM Fe(II), 200 mM ascorbate, 2 mM 2OG, 60 nM HIF1α-CODD peptide (residue 554–574), and inhibitor DMSO (2% DMSO final concentration). Inhibitors were pre-incubated with PHD2 (15 mins) before incubation (10 mins). (**E**) Immunoblotting amifostine/WR-1065 (6 hours, Hep3b cells) treatment. (**F**) Immunoblotting after amifostine/WR-1065 (6 hours, RCC4 cells) treatment. (**G**) Immunoblotting after amifostine/WR-1065 (15/30/60 mins) treatment. (**H**) Immunoblotting of prolyl-hydroxylated HIF1α after amifostine/WR-1065 (15/30/60 mins) treatment.

The above results suggest a depletion of oxygen via consumption by amifostine, inhibition of uptake by mitochondria and inefficient electron transport or direct inhibition of prolyl hydroxylases. To investigate whether the effect of HIF stabilization after administration of amifostine might result from the latter, we performed *in vitro* binding experiments and inhibition assays with a recombinant form of the catalytic domain of PHD2^21^. NMR analysis^13^ (Fig. 4A–C) showed that amifostine does not bind to PHD2 whereas the active metabolite of amifostine WR-1065 is a weak binder. PHD2 hydroxylation assays (Fig. 4D) also showed no evidence of PHD2 inhibition with either amifostine or WR-1065 at up to 200 μM. Cell-based results in other cancer cell lines similarly showed no induction of HIF in Hep3b (Fig. 4E) cells or reduced HIF hydroxylation in RCC4 cells (Fig. 4F) after 6 hours of amifostine or WR-1065 treatment.

Since the response of HIFα induction in the normal hepatocytes is quite short-lived, the experiment was repeated with a higher dose of Amifostine/WR-1065 for a shorter duration. Interestingly, neither amifostine nor WR-1065 were able to induce HIF1α (Fig. 4G) or reduce the level of HIF1α hydroxylation (Fig. 4H) at 250 μg/ml within 1 hour in these two cell lines and conditions. As we show a rise in O_2_ in peripheral blood, a possible mechanism involves a reduction in oxygen consumption by mitochondria, with consequent modulation of ROS production, which impacts on PHD activity.

Taken together, these results show that observation of transient HIF stabilization and upregulation of target genes is unlikely to be due to inhibition of the HIF prolyl hydroxylases resulting from direct binding by amifostine or WR-1065. One possibility is that amifostine mediated delivery of the thiol amifostine WR-1065 into normal liver cells results in a sharp intracellular oxygen consumption as thiol to disulfide conversion occurs, leading to reduced PHD activity. The amifostine mediated hypoxic effect is temporary as the amount of WR-1065 is dose limited and homeostasis mechanisms counteract its effects.

### Effect on cell viability and radio-sensitization and role of HIF

**Figure 5:**
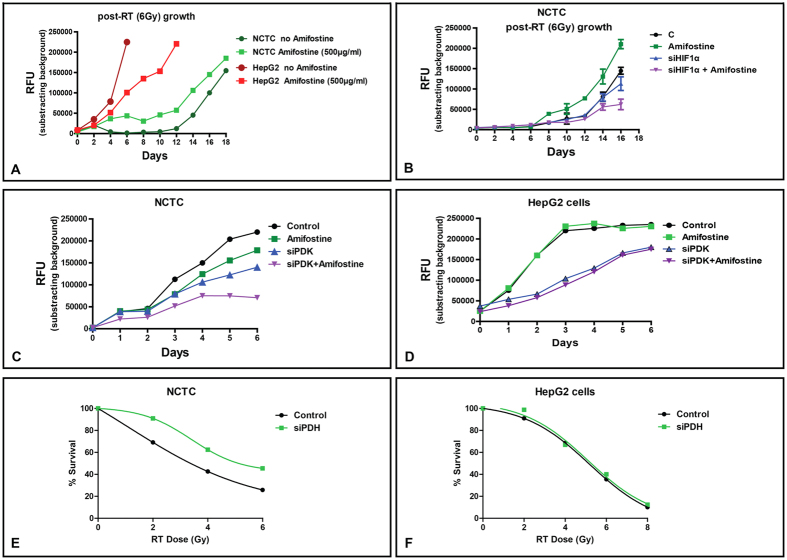
(**A**) Growth kinetics of NCTC liver and HepG2 hepatoma cells following irradiation with 6Gy, with and without 1 h pre-treatment with amifostine (100 μg/ml). **(B)** Growth kinetics of NCTC hepatocytes following silencing of the PDK1 gene, with and without 1 h pre-treatment with amifostine (100 μg/ml). **(C**,**D)** Growth kinetics of NCTC and of HepG2 hepatoma cells following silencing of the PDK1 gene, with and without 1 h pre-treatment with amifostine (100 μg/ml). **(E**,**F)** Radiation dose-response curves of NCTC and of HepG2 cells following silencing of the PDH gene.

The cytoprotective effect of amifostine must be, at least partially, selective to have clinical utility. Further, if amifostine reaches the tumor environment *in vivo* it should not affect the already hypoxic cancer cells. However, there is still activity of the Krebs cycle in cancer cells, providing up to 50% of ATP in better oxygenated areas, so it was important to investigate this population. Amifostine had no effect on the hepatoma HepG2 cell metabolic profile. Normal liver and hepatoma cells were incubated for 1 h with 100–500 μg/ml of amifostine and then irradiated with 6Gy. Cell viability was recorded every two days, and confirmed a substantial protection of normal liver cells. In contrast, hepatoma cells had a poorer viability when amifostine was added prior to irradiation (Fig. 5A). We, thereafter, blocked HIF1α expression in NCTC cells and repeated the same experiment. Notably, amifostine appeared to protect NCTC cells, only when HIF1α expression was sustained (Fig. 5Β).

We then examined the combined effect of amifostine (1 h incubation at 100 μg/ml) with silencing PDK1 gene expression on the growth of NCTC and HepG2 cells. In NCTC cells siPDK1 resulted in reduction of cell growth, while combination with amifostine further suppressed cell growth, suggesting other HIF targets may also contribute to its effect (Fig. 5C). In contrast, although siPDK per se resulted in a profound suppression of HepG2 viability, addition of amifostine (1 h incubation at 100 μg/ml) did not enhance the effect (Fig. 5D). We further assessed if silencing pyruvate dehydrogenase PDH, an effect postulated to mimic amifostine’s activity, influenced NCTC or HeG2 radio-sensitivity. Indeed, silencing PDH resulted in increased resistance of NCTC cells to radiation (Fig. 5E), whilst this had no effect on neoplastic HepG2 hepatoma cells (Fig. 5F).

The shift of metabolism of a normal tissue to aerobic glycolytic and HIF driven pathways, leads inevitably to decreased glucose concentration in the tissue vasculature. As any tumor grows within a normal tissue, such an effect of amifostine should result in reduced glucose accessibility for cancer cells. This in fact may sensitize cancer cells to irradiation, while at the same time normal tissues are protected by switching to HIF mediated metabolism and consuming glucose more avidly. Several clinical data have reported better clinical outcome after radiation in patients receiving amifostine^22^.

## Conclusions

The results reveal that amifostine, following its dephosphorylation in liver tissue, induces PDH-kinase expression via HIF activation, possibly via reduced PDH2 activity. Administration of amifostine to humans and experimental animals reduces the ability of normal liver to consume oxygen, while at the same time glucose consumption is increased. This latter effect is supported by overexpression of glucose transporters and of LDH5 mediated glycolysis. The mechanism by which amifostine induces HIF1 will need further investigation, but seems likely to involve normal tissue mitochondria changes in membrane potentials, oxygen consumption and ROS production. The finding that amifostine sensitizes hepatoma cells to radiotherapy, in contrast to normal hepatocytes, should be considered in clinical protocols recently developed to treat liver primary and metastatic tumors with modern radiotherapy techniques^23^.

## Materials and Methods

### Patients

A total of 74 breast cancer patients were recruited in the study. Twenty of these patients were randomized for basal metabolic rate assessment before and after injection of amifostine or of placebo (water for injection). Thirty patients were randomized for venous glucose level measurements before and after injection of amifostine or of placebo (water for injection). Twenty-four patients were assessed for venous lactate levels, before and after injection of amifostine or of placebo (water for injection) (non randomized cohort).

All patients had no evidence of local disease or distant metastasis and had been referred for post-operative radiotherapy after complete excision of the primary tumor and axillary node dissection. Patients with persistent local disease or evidence of metastasis, diabetes or thyroid disease were excluded from the protocol. Patients with active infection, pregnancy or other known active systemic disease were also excluded. The median age of patients was 59 years (range 35–74). All patients gave written informed consent. The study was approved by the Institute Ethics and Scientific Committee (PGNA D.S.32^ο^/6-9-2007). All clinical studies were carried out in accordance with the World Medical Association Declaration of Helsinki (Bulletin of the World Health Organization, 2001).

### Assessment of the basal metabolic rate (BMR)

Oxygen (O_2_) consumption and carbon dioxide (CO_2_) production was assessed in 20 patients with breast cancer by an indirect calorimetric canopy metabolic test, using the Vmax^®^ system (VIASYS Healthcare GmbH Germany). Before each measurement the system was prepared and calibrated according to the manufacturers’ instructions. Patients were asked to rest in the supine position in an isolated room connected to the canopy system. BMR started after 15 min of rest. The BMR was performed for 25 min. Thereafter, patients received subcutaneously 500 mg of amifostine in 2.5 ml water for injection Winj (10 pts) or Winj alone (10 pts; placebo group). BMR was recorded for 25 minutes. The difference of oxygen consumption (VO_2_ after – VO_2_ before: dVO_2_) and of CO_2_ production (VCO_2_ after – VCO_2_ before: dVCO_2_) between the two halves of recording was calculated for each patient. The randomization procedure was made according to the order of admission in the study (1:1). There was no significant difference in terms of age or body mass index between randomized cohorts [amifostine vs. placebo (mean ± standard deviation): age 58 ± 11 vs. 56 ± 13 years/p-value = 0.73; weight 72 ± 19 vs. 78 ± 14 Kg/p-value = 0.47; height 158 ± 10 vs. 161 ± 7 cm/p-value = 0.42].

### Assessment of glucose and lactate blood levels

Peripheral venous blood glucose levels were assessed in two additional cohorts of 15 breast cancer patients each, at 0 min, 45 min and 24 h, following injection of 500 mg of amifostine diluted in 2.5 ml Winj or Winj only (placebo). Glucose was assessed in a blood drop of patients after puncturing the tip of the index finger, using the Precision Xceed^®^ apparatus (Abbott Diabetes Care Ltd., Oxon, UK). The randomization procedure, as above, was made according to the order of admission in the study (1:1). There was no significant difference in terms of age or body mass index between randomized cohorts [amifostine vs. placebo (mean ± standard deviation): age 59 ± 12 vs. 58 ± 10 years/p-value = 0.83; weight 74 ± 12 vs. 78 ± 17 Kg/p-value = 0.51; height 159 ± 6 vs. 156 ± 9 cm/p-value = 0.34].

Using a similar procedure and the ^‘^Accutrend Lactate^®^’ apparatus (Hoffmann-La Roche Ltd., Basel, Switzerland), lactate was measured in two additional cohorts of breast cancer patients at 0 min and 30 min (12 pts) and, at 0 min and 45 min (12 pts), following injection of 2.5 ml of Winj (placebo). The day after, similar measurements were performed in the same patient, using amifostine (500 mg diluted in 2.5 ml Winj) instead of placebo. In this study, although patients were not randomized, all received placebo on the first day and amifostine on the second and each patient functioned as control for herself.

Blood samples were obtained early in the morning 8–9 am and patients were instructed not to eat or drink any food (but water) after midnight of the previous day.

### Mouse experiments

Animal care and handling was carried out according to the guidelines set by Directive 86/609/EEC. All experimental procedures were approved by the Veterinary Direction for Animal Research in the Department of Experimental Surgery (Protocol No 1610/1-2-13), at the Democritus University of Thrace. All animal experiments were carried out in accordance with the Guide for the Care and Use of Laboratory Animals (The National Academies Press, 2011) and all efforts were made to minimize animal number and their suffering. Approval has been also granted by the Democritus University of Thrace Research and Ethics Committee (Protocol No 035/20-6-13). Male and female mice (Balb/c) 14 to 16 weeks of age (33 ± 2 gr), were under normal conditions concerning ambient temperature (21–23 °C), diet, tap water *ad libitum* and were maintained on a 12 h light: 12 h dark cycle.

Three groups of mice (two male and one female in each one) were recruited as follows *A*. Control group: three mice received subcutaneously injection of Winj and were sacrificed 30 min later *B*. 30 min group: three mice were subcutaneously injected with 200 mg/kg of amifostine in equal volume of Winj and sacrificed 30 min later and, *C*. 60 min group: three mice were subcutaneously injected with 200 mg/kg of amifostine in Winj and sacrificed 60 min later. The sampled liver was snap frozen in liquid nitrogen and stored in −80 °C. In this way we had two male and one female mouse for western blot analysis, for the three time points.

Liver tissues were fragmented on ice with a scalpel. For every mg of tissue sample, 0.015 ml of extraction buffer [10 ml Ripa Buffer (Sigma-Aldrich, R0278), 1 tablet of Complete Protease Inhibitor Cocktail Tablets (Roche), 0.1 ml Phosphatase Inhibitor Cocktail 2 (Sigma, P5726), 0.1 ml Phosphatase Inhibitor Cocktail 3 (Sigma, P0044)] was added and homogenization took place. When homogenization was completed, 2 volumes of acetone per 1 volume of sample were added and the samples were centrifuged 3 times at 12,000 rpm for 3 min. Thereafter, the supernatant acetone was discarded, 1 ml of dH_2_O was added, centrifuged at 12,000 rpm for 3 min and supernatant was discarded again. In the protein pellet, 0.7 ml of Laemmli sample buffer [100 mM Tris pH6.8, 2% w/v SDS (Applichem, A0676), 2% v/v β-mercaptoethanol, 20% v/v glycerol (Sigma, G5516), 0.001% Bromophenol Blue and 36% v/v dH_2_O] were added and further homogenization carried out. The samples were stored at −20 °C. Total whole fraction protein concentrations were estimated according to the Lowry method.

### Normal liver and hepatoma cell cultures

NCTC cells (NCTC clone 1469; CLS) were cultured in culture medium DMEM (Gibco^®^) supplemented with 10% Horse Serum (Gibco^®^) and 1% L-Glutamine (200 mM, Gibco^®^) at 37 °C, 5% CO2. HepG2 (human epithelial hepatocellular carcinoma) (ATCC HB-8065) were maintained and cultured in DMEM-Low Glucose medium (LM-D1102/500, Βiosera), supplemented with 10% Foetal Bovine Serum (FB-1000/500, Βiosera), 2 mM L-glutamine (25030, Gibco), 100 U/ml penicillin, and 100 μg/ml streptomycin (15140-122, Gibco) at standard conditions, 37 °C, 5% CO_2_ in humidified atmosphere and were used upon reaching 70–90% confluency.

Cells were seeded for 24 h in petri dishes (10^6^ cells/per petri). After 24 hours, the cells were treated with 100 and 500 μg/ml amifostine (Ethyol^®^) for 30 min and 1 h. For immunoblotting, whole-cell lysates from cell line were prepared in RIPA buffer (Sigma-Aldrich, cat. no. R0278) with the complete mini protease inhibitor cocktail (Roche Diagnostics, GmbH) and phosphatase inhibitor cocktail (Cell Signaling Technology Inc.). Total protein was estimated using the Pierce™ BCA™ Protein Assay (Thermo Scientific).

### Western blot analysis

For the immunoblotting analysis we used PVDF-PSQ membranes (0,4 mm pore size, Millipore Corp., ISEQ00010). Proteins were separated at 10% SDS separating gel and transferred at 50 V for 90 min. The membrane was blocked using 5% non fat dry milk diluted in TBST (150 mM NaCl, 19 mM Tris, pH 7.5) at room temperature for 2 hrs. The membranes were incubated overnight at 4 °C with the appropriate primary antibody. Membranes were then incubated for 2 h at room temperature with the appropriate secondary antibody. Finally, the membranes were developed with ECL using the ChemiDoc^®^ MP System (Biorad Laboratories Inc.).

For HIF1α detection we used the mouse monoclonal antibody ESEE 122 (Oxford, UK) 1:100. The secondary antibody we used was the rabbit-anti-mouse polyclonal IgG antibody (NB-720, Novus Biologicals,1:50000).

For glucose transporter 2 (GLUT2) protein detection we used the rabbit polycloncal antibody ab54460 (Abcam, UK; 1:5000) raised against a synthetic peptide conjugated to KLH corresponding to amino acids in the cytoplasmic domain of rat Glucose Transporter GLUT2. GLUT2 is the typical transporter of glucose in adult hepatocytes. The secondary antibody we used was the rabbit-anti-mouse polyclonal IgG antibody (NB-720, Novus Biologicals,1:50000).

For lactate dehydrogenase 5 (LDH5) detection we used the sheep polyclonal ab9002 antibody raised against human lactate dehydrogenase V, purified from human placenta (Abcam, UK; 1:5.000). The LDH5 isoenzyme is composed of 4 M (muscle)-subunits and has the highest potency in catalyzing the transformation of pyruvate to lactate a reaction characteristic of the anaerobic glycolysis. For lactate dehydrogenase 1 (LDH1) we used the sheep polyclonal ab9001 antibody (Abcam, Cambridge, UK; 1:5.000), raised against human LDH-I purified from erythrocytes. The LDH1 isoenzyme is compose by 4 H (heart)-subunits and favors the reverse transformation of lactate to pyruvate, fueling the oxidative phosphorylation. The secondary antibody used was the rabbit-anti-sheep polyclonal IgG antibody (1:50,000; Dako, PO163).

For the study of pyruvate dehydrogenase kinase (PDH-Kinase or PDK) we used the mouse monoclonal ab110025 anti-mitochondrial Pyruvate Dehydrogenase Kinase 1 antibody monoclonal primary anti-mouse antibody (ab110025, ABCAM; 1:2500). PDK1 is a key enzyme that phosphorylates PDH, leading to inactivation and blockage of production of Acetyl-CoA from pyruvate, thus suppressing the mitochondrial aerobic metabolic pathway. The secondary antibody we used was the rabbit-anti-mouse polyclonal (NB-720, Novus Biologicals, 1:10.000).

For pyruvate dehydrogenase PDH detection we used the ab110333 mouse monoclonal antibody (ABCAM, UK, 1:2500) raised against porcine Pyruvate Dehydrogenase E2/E3bp was used. The secondary antibody we used was the polyclonal rabbit-anti-mouse (NB-720, Novus Biologicals, 1:10.000).

For the phosphorylated (inactive) form of pyruvate dehydrogenase (PDH) we used the rabbit polyclonal ab92696 antibody raised against the anti-Pyruvate Dehydrogenase E1-alpha subunit (phospho S293) (ABCAM, UK, 1:2500). This antibody recognizes the phosphorylated, thus the inactive, form of PDH. The secondary antibody we used was the goat-anti-rabbit polyclonal IgG antibody (NB-730, Novus Biologicals, 1:10.000).

For human HIF1α detection, the following antibodies were used: pan-anti-HIF-1α (clone 54, BD Transduction Laboratories™, 1:1000), anti-HIF-1α Hyp402 (catalogue number 07-1585, Merck Millipore, 1:500), anti-HIF-1α Hyp564 (D43B5, Cell Signaling, 1:1000), anti-HIF1α HyN803 (1:2000), and HRP-conjugated anti-β-actin (clone AC15, Abcam).

The images of the blots were captured utilizing Chemidoc MP imaging system (Biorad, USA) and band densitometry analysis was performed using the Chemidoc-logismic.

### Confocal immunofluorescence microscopy

For immunofluorescence staining, mouse liver tissue cryo-sections, of control untreated animals, treated with amifostine for 30 minutes and 60 minutes respectively (2 μm) were fixed in ice-cold methanol for 10 min at −20 °C. In case of the NCTC mouse liver cell line, cells grown on glass coverslips #1.5 were treated according to the experimental conditions, fixed in 3.7% formaldehyde/PBS for 20 minutes at room temperature and then permeabilized in PBS/0.1% v/v Triton X-100 pH 7.4 for 5 minutes. Both tissues sections and treated cells grown on coverslips were blocked in PBS/3% w/v BSA pH 7.4 and stained with various combinations of the above mentioned antibodies: anti-PDH mouse monoclonal (1:1000), anti P-PDH rabbit polyclonal (1:500), anti-GLUT2 rabbit polyclonal (1:100), anti-PDK mouse monoclonal (1:200), anti-LDHV sheep polyclonal (1:200), anti-LDHI sheep polyclonal (1;200) and anti-HIF1α mouse (1:20), for 1 h at room temperature. The samples were washed in PBS pH 7.4, incubated with appropriate CF 488, 568 and 640 secondary antibodies (1:500; Biotium) for 30 min a room temperature and DNA was counterstained with Hoechst 33342 (1 μg/ml; Sigma-Aldrich). After final washes coverslips were mounted on the top of slides using homemade Mowiol mounting medium.

Imaging of fixed samples was performed using a customized Andor Revolution Spinning Disk Confocal System built around a stand (IX81; Olympus) with a 60x- lens and a digital camera (Andor Ixon + 885) (CIBIT Facility, MBG-DUTH). Image acquisition was performed in Andor IQ 2 software. Optical sections were recorded every 0.3 μm.

Fluorescence intensity analysis for five representative confocal microscopy images was performed using Image J 1.47v (National Institute of Health, USA) software. Image processing macros were developed in order to quantify the levels of the examined protein in the area of interest. Graph presentation was performed using the GraphPad Prism Version 5.01a statistical package (GraphPad Software Inc., USA).

### Acetyl-CoA assay

Acetyl-CoA concentration in whole fraction lysate from NCTC cells in the presence of 100 μg/ml Amifostine at two time- points of treatment, for 30 and 60 minutes respectively, was measured by a fluorescence assay compared to untreated cells, using a commercial kit (ab87546; Abcam, UK) according to the manufacturer’s instructions. Following scraping of the cells, samples were sonicated 4 times for 5 seconds each, on ice, and microcentrifuged for 10 minutes at 4 °C. The supernatant/ cell lysate was then transferred into a new tube. Following a deproteinization step with perchloric acid and KHCO_3,_ 10 ul of each sample was added in duplicate to subtract the background, in which the acetyl-CoA was not converted in CoA and then to NADH, to finally generate the fluorescent product with PicoProbe. Reaction mixtures were incubated at 37 °C for 10 minutes, fluorescence measured at Ex/Em = 535/589 nm in Fluo Star (Omega). A standard curve was generated in the 0–40 pmol range, with 10 pmol/well increments, in order to calculate the acetyl-CoA concentration in each condition.

### ATP measurement

The ATP concentration of NCTC cells in the presence of 100 μg/ml Amifostine at two time- points of treatment, for 30 and 60 minutes respectively, was measured by a luminescence assay compared to untreated cells, using the ViaLight^TM^ plus kit, (Lonza). Briefly, cells were plated in a luminescence compatible 96 well plate and 24 h later amifostine was added. After the incubation time points, medium was removed and 100 μl of fresh medium was added. Then, the following steps prior to measurement were followed according to the manufacturer instructions; the 96-well plate was removed from the incubator and left to equilibrate at room temperature for at least 5 min. 50 μl of cell lysis reagent was then added to each well and the plate were left for a further 15 min incubation at room temperature. Finally, 100 μl of AMR plus assay buffer were added and a 2 min final incubation at room temperature carried out. Luminescence was measured utilizing the Omega Fluostar machine (BMG Labtech).

### Monitoring mitochondrial membrane potential with live-imaging

For mitochondrial immunofluorescence staining and live imaging a JC1 mitochondrial membrane potential assay kit (Abcam) was used according to the manufacturer’s instructions, NCTC mouse liver cells were grown to ~90% confluency in μ-Slide 4 Well, ibiTreat slides: #1.5 polymer coverslip, tissue culture treated, sterilized (Ibidi). The mitochondrial membrane potential (Δψm) kit uses JC1, tetraethylbenzimida-zolylcarbocyanine iodide, a cationic dye has been used by mitochondrial membrane potential kit, given accumulates in energized mitochondria to measure the mitochondrial membrane potential. In details, at low concentrations (due to low Δψm) JC-1 is predominantly a monomer with a green fluorescence (emission of 530 ± 15 nm) while at high concentrations (due to high Δψm) the dye aggregates yielding a red to orange fluorescence (emission 590 ± 17.5 nm). NCTC untreated (control cells), amifostine treated cells (100 μg/ml) and FCCP, a depolarization positive control treated cells (100 μM) were been monitored at 37 °C in an appropriate live imaging chamber by confocal microscopy. The FCCP [carbonyl cyanide 4-(trifluoromethoxy) phenylhydrazone] is an ionophore uncoupler of oxidative phosphorylation, cells treatment with FCCP eliminates mitochondrial membrane potential and JC1 staining. Living imaging was performed on a customized Andor Revolution Spinning Disk Confocal System built around a stand (IX81; Olympus) with a 100x-1.4 NA lens and a digital camera (Andor Ixon + 885) (CIBIT Facility, MBG-DUTH). Image acquisition was performed in Andor IQ 2 software. Optical sections were recorded every 0.3 μm. All confocal microscopy images presented in this work are 2D maximum intensity projections of z-stack images, and image analysis and quantification of the obtained data sets has been performed using ImageJ 1.47v (National Institute of Health, USA). A population of n cells (n ≥ 50) has been analyzed for various time points (0, 5, 10, 15 and 20 minutes respectively), while time-lapse confocal microscopy has been used for the 0–5 minutes interval. Graph presentation has been performed using the GraphPad Prism Version 5.01a statistical package (GraphPad Software Inc., USA).

### Monitoring mitochondrial superoxide production

For mitochondrial superoxide staining and live imaging, the Mito-SOX Red mitochondrial superoxide indicator for live cell imaging (Molecular probes by Life Technologies) was used according to the manufacturer’s instructions, NCTC mouse liver cells and HepG2 human hepatocellular carcinoma cells were grown to ~90% confluency in μ-Slide 4 Well, ibiTreat slides: #1.5 polymer coverslip, tissue culture treated, sterilized (Ibidi). Superoxide production by mitochondria was visualized by confocal microscopy using the MitoSOX™ Red reagent. MitoSOX™ Red reagent enters live cells where it selectively targets mitochondria, then it is quickly oxidized by superoxide but not by other reactive oxygen species (ROS) and reactive nitrogen species (RNS). The final oxidized product is highly fluorescent upon binding to nucleic acid and can be imaged using a red laser and a 561 nm emission filter.

Untreated (control cells), amifostine treated cells (100 μg/ml), irradiated cells (18Gy) and amifostine treated irradiated cells (18Gy) and have been monitored at 37 °C in an appropriate live imaging chamber by confocal microscopy. Living imaging was performed using a customized Andor Revolution Spinning Disk Confocal System built around a stand (IX81; Olympus) with a 100x-1.4 NA lens and a digital camera (Andor Ixon + 885) (CIBIT Facility, MBG-DUTH). Image acquisition was performed using the Andor IQ 2 software. Optical sections were recorded every 0.3 μm. All confocal microscopy images presented in this work are 2D maximum intensity projections of z-stack images, and image analysis and quantification of the obtained data sets has been performed using ImageJ 1.47v (National Institute of Health, USA).

### Viability experiments

NCTC and HepG2 cells were harvested and seeded at a density of 250 cells/well in a 96-well plate. Irradiation of the plates was performed using a 6 MV beam of a Linear Accelerator (PRECISE; ELEKTA) equipped with a MultiLeaf collimator. For multidose irradiation of the well columns within the same 96-well plate, a previously validated and reported technique was used. Irradiation experiments were performed in control cells and cells pretreated for 1 h with 100–500 μg/ml of amifostine. Cell viability and survival experiments were performed using the AlamarBlue^®^ and the CyQUANT^®^ cell viability assays, as previously validated by our group. The RFU (Relative Fluorescence Units) were measured using OMEGA fluostar multiwell plate reader (BMG Labtech).

Time course cell growth experiments were also performed after exposure of cells to amifostine and/or after silencing of the PDK1 gene. PDK siRNAs were custom synthesized (GenePharma Co; China), pooled and used at 100 nM to transfect cells using Metafectene^®^ Pro (BIONTEX) for 24 h; the silencing efficiency of siRNAs was confirmed both by western blot after 48 h in total. After 48 h from the silencing, cells were placed in 96-well plates at a concentration of 5000 cells per well and incubate with 100–500 μg/ml Amifostine (Ethyol^®^) for 1 h. Cell density was daily recorded with the above described methods.

### Quantitative Real-Time PCR

Three groups of 3 (three) male Balb/c mice each were sacrificed and livers were excised before and 15 and 60 minutes after the subcutaneous injection of 200 mg/kg of amifostine (3 mice in each time point). Tissues were stored in RNA later solution (AM7020, Ambion, France) at −80 °C. Nucleospin RNA plus (740984, Macherey-Nagel, Germany) was used for RNA isolation from tissues. Although, this kit removes gDNA using removal columns we tested the samples for gDNA contamination with 2% w/v gel agarose. Then, 1 μg of extracted RNA was employed for first strand cDNA synthesis using Transcriptor First Strand cDNA synthesis kit (04379012001, Roche, Switzerland).

PDK1, LDHA, GLUT1 and β-actin genes were amplified in duplicates using a Light Cycler 480II (Roche, Switzerland) and Sensimix SYBR No-ROX kit (QT650, Bioline, USA). The PCR reaction mixture was prepared at 20 μl final volume, consisted of 10 μl 2x Sensimix SYBR NO-ROX, 0.5 μl of 10 μΜ of each primer and 1 μl of template. The PCR reaction was initiated with a preheated step at 95 °C for 15 sec followed by 40 cycles of denaturation step for 15 sec at 95 °C, annealing step at 55 °C for 15 sec and extension step at 72 °C for 15 sec. Primer sets were designed using Universal ProbeLibrary Assay Center (Roche, France). Dissociation curve and 2% gel agarose were performed for specificity of the products to be verified. β-Actin was used as housekeeping gene and alterations in gene expression were expressed as 2^−ΔΔCt^ fold-change in mRNA levels.

### NMR experiments

Nuclear Magnetic Resonance (NMR) spectra were recorded using a Bruker AVIII 600 MHz NMR spectrometer equipped with a BB-F/^1^H Prodigy N_2_ cryoprobe using 5 mm diameter NMR tubes (Norell) as reported^24^,^25^. A recombinant form of the catalytic domain of human PHD2 (residues 181–426) was used in all NMR experiments. All experiments were conducted at 277 K. Data were processed with Bruker 3.1 software. Prior to Fourier transformation, data were multiplied with an exponential function of 3 Hz.

#### ^1^H NMR experiments

90^o^ pulse lengths were 11.03 μs and spectra were typically obtained with 64 scans using a relaxation delay of 1 s and a pre-scan delay of 10 μs. A 2 ms sinc pulse was used for water suppression. The assay mixtures contained 50 μM *apo*-PHD2 supplemented with 200 μM Zn(II) and 500 μM of Amifostine or its active metabolite (WR-1065) buffered with 50 mM Tris-D_11_ (pH 7.5) and 0.02% NaN_3_ in 90% H_2_O and 10% D_2_O.

#### wLOGSY NMR experiments

Water-Ligand Observed Gradient SpectroscopY (wLOGSY) experiments were conducted using the pulse sequence described by Dalvit *et al*.^26^. Unless otherwise stated, all experiments were conducted at 277 K. Typical experimental parameters were as follows: mixing time, 1 s; relaxation delay, 2 s; number of transients, 256. Solvent excitation was achieved using a 16 ms 180 degree selective rectangular shape pulse with 100% truncation level and 1000 points (Squa100.1000) set at the H_2_O frequency. Water suppression was achieved by a Sinc1.1000 pulse at the H_2_O frequency. The assay mixtures contained 50 μM *apo*-PHD2 supplemented with 200 μM Zn(II) and 500 μM of Amifostine or its active metabolite (WR-1065) buffered with 50 mM Tris-D_11_ (pH 7.5) and 0.02% NaN_3_ in 90% H_2_O and 10% D_2_O.

#### ^1^H CPMG NMR experiments

Typical experimental parameters for Carr-Purcell-Meiboom-Gill (CPMG) NMR spectroscopy were as follows: total echo time, 40 ms; acquisition time, 2.72 s; relaxation delay, 2 s; number of transients, 1024. The PROJECT-CPMG sequence (90°x−[τ−180°y−τ−90°y−τ−180°y−τ]n−acq) as described by Augilar *et al*.^27^ was applied. Water suppression was achieved by pre-saturation. Data were collected with a sweep width of 12019 Hz and an acquisition time of 2.7 s. Assay mixtures contained equimolar amounts (100 μM) of *apo*-PHD2 and amifostine or its active metabolite (WR-1065) supplemented with 200 μM Zn(II) and and 0.02% NaN_3_ in 90% H_2_O and 10% D_2_O, 50 mM Tris-D_11_ (pH 7.5).

#### AlphaScreen^®^ PHD2 hydroxylation Assay

The AlphaScreen^®^ PHD2 hydroxylation assays were carried out in 384-well white ProxiPlates™ (PerkinElmer) as reported^28^. Reactions were performed in buffer containing 50 mM HEPES pH 7.5, 0.01% Tween-20 and 0.1% BSA in a final volume of 10 μM at room temperature. A mixture of 5 nM PHD2 (residues 181–426), 20 μM Fe(II), and 200 μM ascorbate was incubated with inhibitors (supplemented with DMSO 2% final concentration) for 15 minutes prior to incubation (10 minutes) with the substrate mixture (60 nM biotinylated CODD peptide-HIF-1α residues556-574) and 2 μM 2OG). The reactions were then quenched with 5 μL 30 mM EDTA. 5 μL of pre-incubated donor-acceptor bead mix (AlphaScreen^®^ streptavidin-conjugated donor and ProteinA-conjugated acceptor beads; PerkinElmer) with HIF-1α hydroxy-Pro546 antibody (3434S, Cell Signaling) were then added to the reaction mixture for 1 hour in the dark at room temperature. The luminescence signal was measured using an Envision (Perkin Elmer) plate reader. Data were analysed utilizing Origin 9.1 from OriginLab^®^.

### Statistical analysis

Statistical analysis was performed using the GraphPad Prism 5.0 (GraphPad Software Inc., USA). The unpaired two-tailed t-test was or the Wilcoxon matched pairs test were used to compare groups with continuous variable data, as appropriate. One way ANOVA non parametric analysis (Kruskal-Wallis test) was also performed to test differences among groups of continuous variables. A p-value of <0.05 was used for significance.

## Additional Information

**How to cite this article**: Koukourakis, M. I. *et al*. Normal tissue radioprotection by amifostine via Warburg-type effects. *Sci. Rep*. **6**, 30986; doi: 10.1038/srep30986 (2016).
